# Addressing stigma and discrimination at scale: uniting for a common vision while acknowledging local realities

**DOI:** 10.1002/jia2.25893

**Published:** 2022-02-28

**Authors:** Lucy Stackpool‐Moore, Sbongile Nkosi, Princess Kasune Zulu, Josie Presley, Laura Ferguson

**Affiliations:** ^1^ IAS Geneva Switzerland; ^2^ Watipa Sydney New South Wales Australia; ^3^ GNP+ Cape Town South Africa; ^4^ Government of Zambia Lusaka Zambia; ^5^ Global Health Division‐HIV Bill and Melinda Gates Foundation Seattle Washington USA; ^6^ Institute on Inequalities in Global Health, University of Southern California Los Angeles California USA

1

A strong evidence base exists about the role of stigma and discrimination in undermining the global HIV response; and yet, urgent action is needed to translate this evidence to address stigma at scale. Too often “stigma and discrimination” are referred to jointly, without due attention to the specific details and action needed to address each distinct yet related area. On Zero Discrimination Day, we acknowledge that stigma and discrimination are due attention and action — independently and jointly — in order to mutually reinforce progress in reducing the harmful effects of stigma and achieving zero discrimination.

Despite decades of scientific advances in prevention and treatment, and widespread awareness‐raising efforts [1], stigma and discrimination remain “twin barriers” impeding progress in response to HIV and are a central concern of the current UNAIDS Global AIDS Strategy to end inequalities. The inclusion of commitments towards eliminating HIV‐related stigma and discrimination within the Political Declaration agreed at the 2021 United Nations High Level Meeting on HIV/AIDS for the first time also signals a conducive global political environment for action at scale. In a similar vein, funders are following the evidence and investing in analyses and programs that have been shown to be effective to address stigma and discrimination, as evinced by the Global Fund's commitment; the attention to intersectional and HIV‐related stigma by the National Institutes of Health; and the investment in consolidating the evidence base and the state of the field by the Bill and Melinda Gates Foundation.

Discrimination can constitute a human rights abuse, for which there are tangible state obligations and accountability measures [2]. Stigma is more subtle and is a process that devalues an individual through labels, which emanate from making people experience “otherness” resulting in social isolation and a diminished sense of self [3]. Stigma and discrimination negatively affect the quality of life of people living with HIV through multiple pathways, including social rejection, low self‐esteem and barriers to accessing health and support services [4]. Healthcare settings can provide lifesaving care, and yet commonly are sites where stigma and/or discrimination may be experienced by people living with HIV, who additionally report devaluing experiences in other social, cultural and institutional settings [5, 6]. Stigma can cause discrimination, entrenched in laws and policies and cultural norms governing peoples’ actions. Stigma can also be triggered by discrimination, as well as other factors, such as anticipation or perception of being judged. Stigma can be internalized, affecting how people feel about themselves and indirectly can impact their actions, which is even more challenging to measure or make “visible” [7, 8].

As defined by the Global Partnership for Action to Eliminate all Forms of Stigma and Discrimination [9]: *HIV‐related stigma* is evident in irrational or fear‐driven negative attitudes, behaviours and judgements towards people living with HIV, their partners and families, and key populations. *HIV‐related discrimination* is unfair and unjust treatment of a person, or group of people, based on their real or perceived HIV status.

Acts of discrimination are often witnessed, documented and verifiable at a specific moment in time or over a series of sustained interactions. As stated by Amon et al., understanding discrimination as a human rights abuse for which governments have specific legal obligations facilitates a more effective response to HIV even in environments with significant, entrenched stigma. Stigma, on the other hand, has been harder to define, measure and ameliorate. As a result, several efforts have attempted to conceptualize and further develop definitions of stigma. Naming and defining domains and drivers of stigma, as well as developing bespoke tools and measurement approaches, have unequivocally finessed concepts and facilitated the development of a robust and diverse evidence base to understand stigma, such as the recent development of the Health Stigma and Discrimination Framework [10]. Other widely cited examples include [11] and [12]. Notably, the People Living with HIV Stigma Index [13 has demonstrated how community leadership and scientific leadership can partner to robustly document stigma and then use a similar tool to track its evolution (or lack thereof) and compare change over time [6]. Yet, an unintended consequence of these many efforts to better theorize and define stigma has been the emergence of a plethora of scales, tools and frameworks tracking and defining stigma differently in different contexts and populations [14]. Concepts of stigma continue to evolve, such as by viewing HIV in relation to other areas, such as sexual orientation and gender identities, age, sex work, illicit drug use and living with a disability [15]. In contrast, discrimination remains “under‐theorized” [2].

Experiences of stigma, and acts of discrimination, exist within life contexts, as well as a larger web of socio‐cultural experiences and inequities. For example, a recent systematic review found that poor health outcomes of adolescents living with HIV were related to anticipated stigma, internalized stigma and stigma experienced when accessing healthcare [16]. Similarly, a more comprehensive view of gender identities has increased, and studies have highlighted stigma experienced by cis‐ and transgender people [17, 18]. HIV‐related stigma and acts of discrimination are hard to disentangle from values and judgements relating to sexuality, age, gender norms and intimate relations. Community‐led interventions often succeed because they seek to achieve this and take a more holistic and peer‐based approach [19].

That stigma is socially constructed, discrimination is legally constructed and both are context specific has complicated our efforts to understand them. Efforts to address stigma, and discrimination, are best situated within multi‐sectoral as well as specific efforts to promote complete health and wellbeing for people living with and most vulnerable to HIV. Everyone is entitled to the highest attainable standard of health defined as physical, mental and social wellbeing; it is a human right.

We must unite and learn from historical efforts, celebrate the courageous individuals and interventions that effectively challenge stigma and seek redress for discrimination, and move forward by better documenting processes, evaluating progress, measuring change over time and learning so that the context specific can also inform a broader view. This requires precise articulation of stigma, and discrimination, in relation to and distinct from each other. Many sectors, including, but not only, health, need to align and address issues that impede quality of life, including manifestations of stigma and acts of discrimination. School curricula and professional education should also focus on solutions, and should be resourced to systemically address inclusion, diversity, respect and dignity for all. Action must be duly targeted, and resources appropriately allocated, so that reducing stigma and achieving zero discrimination can be realized at scale.

## COMPETING INTERESTS

LSM, SN, PKZ and LF declare no competing interests. JP works for the Bill and Melinda Gates Foundation, which supports the “getting to the heart of stigma initiative” of the IAS.

## AUTHORS’ CONTRIBUTIONS

LSM, LF, JP, SN and PKZ conceptualized the argument. LSM wrote the draft. LF, JP, SN and PKZ commented and reviewed the viewpoint. LSM finalized the viewpoint.
