# Corrigendum

**DOI:** 10.1002/jia2.25940

**Published:** 2022-05-28

1

In the article [1], there was an error made in the 5^th^ paragraph of body text.

The last sentence reads: “In contrast, discrimination remains “under‐theorized” [2].”

The last sentence should have read: “In contrast, discrimination remains “under‐theorized” among stigma researchers [2].”
